# Disrupted Sleep Homeostasis and Altered Expressions of Clock Genes in Rats with Chronic Lead Exposure

**DOI:** 10.3390/toxics9090217

**Published:** 2021-09-10

**Authors:** Chung-Yao Hsu, Yao-Chung Chuang, Fang-Chia Chang, Hung-Yi Chuang, Terry Ting-Yu Chiou, Chien-Te Lee

**Affiliations:** 1 Department of Neurology, Kaohsiung Medical University Hospital, Kaohsiung 80708, Taiwan; cyhsu61@gmail.com; 2 Department of Neurology, School of Medicine, College of Medicine, Kaohsiung Medical University, Kaohsiung 80708, Taiwan; ycchuang@cgmh.org.tw; 3 Department of Neurology, Kaohsiung Chang Gung Memorial Hospital, Kaohsiung 83301, Taiwan; 4 College of Medicine, Chang Gung University, Taoyuan 33302, Taiwan; tytc107@gmail.com; 5 Institute for Translation Research in Biomedicine, Kaohsiung Chang Gung Memorial Hospital, Kaohsiung 83301, Taiwan; 6 School of Veterinary Medicine, National Taiwan University, Taipei 10617, Taiwan; fchang@ntu.edu.tw; 7 Department of Public Health and Environmental Medicine, College of Medicine, Kaohsiung Medical University Hospital, Kaohsiung Medical University, Kaohsiung 80708, Taiwan; ericch@kmu.edu.tw; 8 Division of Nephrology, Department of Internal Medicine, College of Medicine, Kaohsiung Chang Gung Memorial Hospital, Chang Gung University, Kaohsiung 83301, Taiwan; 9 Chang-Gang Kidney Research Center, Kaohsiung 83301, Taiwan

**Keywords:** chronic lead exposure, sleep homeostasis, clock genes, neurotoxicity, circadian rhythm, sleep–wake cycle

## Abstract

Sleep disturbance is one of the neurobehavioral complications of lead neurotoxicity. The present study evaluated the impacts of chronic lead exposure on alteration of the sleep–wake cycle in association with changes of clock gene expression in the hypothalamus. Sprague–Dawley rats with chronic lead exposure consumed drinking water that contained 250 ppm of lead acetate for five weeks. Electroencephalography and electromyography were recorded for scoring the architecture of the sleep–wake cycle in animals. At six Zeitgeber time (ZT) points (ZT2, ZT6, ZT10, ZT14, ZT18, and ZT22), three clock genes, including *rPer1*, *rPer2*, and *rBmal1b*, were analyzed. The rats with chronic lead exposure showed decreased slow wave sleep and increased wakefulness in the whole light period (ZT1 to ZT12) and the early dark period (ZT13 to ZT15) that was followed with a rebound of rapid-eye-movement sleep at the end of the dark period (ZT22 to ZT24). The disturbance of the sleep–wake cycle was associated with changes in clock gene expression that was characterized by the upregulation of *rPer1* and *rPer2* and the feedback repression of *rBmal1b*. We concluded that chronic lead exposure has a negative impact on the sleep–wake cycle in rats that predominantly disrupts sleep homeostasis. The disruption of sleep homeostasis was associated with a toxic effect of lead on the clock gene expression in the hypothalamus.

## 1. Introduction

Lead, a toxic metal, is one of the most common cumulative and preventable toxic pollutants of our environment that can induce various adverse clinical consequences in children and adults [1,2,3]. As lead can be stored in bones for decades, the toxic effects of lead may be retained throughout the life course [4,5]. Lead and other heavy metals in bones may serve as a source of exposure during different stages of one’s life and have a potential cumulative effect on adverse clinical outcomes [3,5]. In addition, lead may persist in the environment; populations can continue to be exposed in the region where it was previously used [3]. Age, country of birth, education level, gender, ethnicity, income, and occupation have shown significant differences depending on the degree of lead exposure, with higher levels of exposure resulting in worse outcomes [3]. Chronic lead exposure has been recognized to have substantial impacts on the renal, hematological, reproductive, cardiovascular, cerebrovascular, and neurological systems in humans [2,6,7,8,9]. In the central nervous system (CNS), the detrimental neurotoxicity of lead is commonly associated with neuropsychological and neurobehavioral difficulties in intelligence, memory, attention, executive tasks, processing speed, language, visuospatial skills, motor abilities, and mood and affection [2,7,10]. Early life exposure to low levels of lead may affect brain development, particularly in impairment of learning and memory function [1]. High-level lead exposure may result in an increase in the prevalence of brain cancer [11]. Several mechanisms for lead neurotoxicity have been proposed that included disruption of long-term potentiation in the hippocampus [10], hyperphosphorylation of tau protein [12], neuroinflammatory response [13,14], oxidative stress [14,15], mitochondrial dysfunctions [14], abnormal zinc homeostasis [16], deregulation of calcium signaling [17], and abnormal neural transmission and gene expression [1,18]. Moreover, lead-induced damage in the prefrontal cerebral cortex, hippocampus, and cerebellum can lead to a variety of neurodegenerative diseases, such as Alzheimer’s disease, Parkinson’s disease, and schizophrenia [16,19]. Clinically, chronic lead exposure can manifest with fatigue, headache, irritability, speech problems, cognitive and memory impairment, seizure disorders, and even sleep disturbances [2,20,21,22].

Sleep disturbance is one of the neurobehavioral complications of lead toxicity [21,22,23]. The cumulative effect of chronic lead stored in bones throughout the life course may influence sleep architecture [3,21,22,24]. Combined lead and zinc exposure have caused severe sleep disturbance and daytime symptoms in copper smelt workers [24]. Elevated blood lead levels in early childhood are associated with an increased risk for sleep problems and excessive daytime sleepiness in later childhood [21,22]. Chronic lead exposure in early life may change the morphology, cellular density, and relative optical density in the cells of the suprachiasmatic nucleus (SCN) of the hypothalamus [25]. This issue of public and environmental health not only indicates that lead neurotoxicity should be concerned from the stage of infancy and even in the prenatal period [26] but also emphasizes that monitoring the synergistic toxic effects of other heavy metals in the population with a high risk of chronic lead exposure is important [27].

The circadian rhythm of stereotyped complex behaviors among lead-exposed rats showed a depressed response in light periods but an elevated response in dark periods [23]. These findings may indicate that behavioral pattern of the circadian rhythm might be disrupted by chronic lead exposure. Whereas sleep activity is the most important component of neurobehavioral functions, the effect of chronic lead intoxication on the sleep–wake cycle and clock gene expression in the hypothalamus is still unclear. In the present study, we aim to evaluate the impacts of chronic lead exposure on alteration of the sleep–wake cycle in association with changes of clock gene expression in the hypothalamus in a chronic lead-exposed animal model.

## 2. Materials and Methods

### 2.1. Study Design

This study is based on an experimental animal model of chronic lead toxication that aimed to explore the toxic effect of lead in the change of sleep–wake cycle and circadian genes. Experimental procedures in the present study were accomplished in accordance with the guidelines on animal experimentation, endorsed by the constituted research ethics committee of Kaohsiung Medical University, Kaohsiung, Taiwan (the project identification code 97033 was approved by the Institutional Animal Care and Use Committee of Kaohsiung Medical University; approval date on 1 August 2008).

### 2.2. Experimental Animals

Specific pathogen-free male Sprague–Dawley rats (postnatal week six; body weight: 250–280 g) were purchased from BioLASCO Taiwan Co. Ltd. (Taipei, Taiwan) and housed in an environmentally controlled room (22–24 °C; 12:12 h light/dark cycle; lights on at 07:00 am) at the Center for Laboratory Animals at Kaohsiung Medical University. Standard laboratory rat chow and tap water were available ad libitum. All efforts were made to reduce the number of animals used and to minimize animal suffering during the experiment.

### 2.3. Experimental Animal Model of Chronic Lead Toxication

The model of chronic lead poisoning in rats has been well established in our laboratory [28]. The animals with chronic lead exposure consumed drinking water that contained 250 ppm of lead acetate for five weeks. The water regimen of lead acetate solution was to dissolve 125 mg of lead acetate powder into 500 mL of reverse osmosis water. Animals that were fed with deionized water and surgical preparations without chronic lead exposure manipulation served as the control group [28].

### 2.4. Electroencephalography and Electromyography Recordings

Both animal groups received anesthesia via intraperitoneally injection of Zoletil^®^ 50 (30 mg/kg, Virbac, Taipei, Taiwan) that contained tiletamine and zolazepam (1:1), then rats underwent surgical interventions for preparation of recordings of the sleep–wake cycle by electroencephalography (EEG) and electromyography (EMG) on the first day of week five. After anesthesia, the head of the rat was fixed to a stereotaxic headholder (Kopf, Tujunga, CA, USA), and the body of rats were kept at 37 °C by placing them on a heating pad. The procedure of EEG recording and scoring for sleep stages was performed according to previous reports [29,30,31,32]. To perform the EEG recording, three EEG screw electrodes were then implanted on the surface of the cortex with the following coordinates: (1) right frontal (2.0 mm anterior to bregma and 3.5 mm from the midline); (2) right hippocampus (3.5 mm posterior to bregma, 2.5 mm from the midline); and (3) left occipital (1.0 mm posterior to lambda and 3.5 mm from the midline) [29,30,32]. The insulated wires from EEG electrodes were routed to a Teflon pedestal (Plastics One, Roanoke, VA, USA). The Teflon pedestal was then cemented to the skull using dental acrylic (Cranioplasty cement and cyanoacrylate gel, Plastics One, Roanoke, VA, USA). For evaluation of EMG activity during the sleep stage, two electrodes were simultaneously inserted into the posterior neck muscles [29,30]. The surgical wounds of animals were then treated topically with povidone-iodine ointment. Animals were returned to the 12 h light/dark cages individually and the rats were allowed a one-week interval for recovery and then prepared for the further EEG and EMG recordings. The drinking water containing lead acetate was continuously fed to the animals until EEG and EMG recording.

The EEG and EMG recordings for scoring the distribution of the sleep–wake cycle in animals under chronic lead exposure were carried out on the first day of week six [29,30]. In brief, the animals were connected to the EEG and EMG recording apparatus via a flexible tether for 24 h. The signal of body movement was determined by custom-made infrared-based motion detectors (Biobserve GmbH, Bonn, Germany), and the movement activity was converted to a voltage output. The signals from the EEG and EMG electrodes were transmitted to an amplifier (model V75-01; Colbourn Instruments, Lehigh Valley, PA, USA) [29,32,33,34]. These conditioned signals, including EEG, EMG, and body movements, were then converted with 16-bit precision at a sampling rate of 128 Hz (NI PCI-6033E; National Instruments, Austin, TX, USA). The digitalized EEG and EMG waveforms and integrated values for body movement were stored as binary computer files for further analyses as described in our previous reports [29,30,33,34].

Postacquisition judgment of the sleep–wake state was evaluated by visual scoring of 12-s epochs (ICELUS, Professor M. R. Opp, University of Michigan) authored in LabView for Windows (National Instruments, Austin, TX, USA) [29,33,35]. The sleep architecture of animals was categorized to slow wave sleep (SWS), rapid-eye-movement (REM) sleep, and wakefulness according to our previous reports [29,30]. Based on these criteria, SWS is characterized by large-amplitude slow waves and high-power density values within the delta frequency band (0.5–4.0 Hz) during EEG recording [29,30,36]. Moreover, EMG activities showed decreased muscle tone and lack of gross body movements. In the REM sleep, the amplitude of EEG is decreased, the density of EEG mainly occurs in the theta range (6.0–9.0 Hz), the activity of EMG often displays muscle atonia and low amplitudes, and phasic body twitches may appear [29,30,36]. In the stage of wakefulness, animals are usually vigorous showing protracted body movement and robust activity and amplitudes in the EMG. The amplitude of the EEG is similar to that observed in REM sleep; however, values of power density in the delta frequency band are usually greater than those in the theta frequency band [29,30,36]. For evaluation of architecture of the sleep–wake cycle, the percentage of SWS, REM sleep, and wakefulness were calculated at each Zeitgeber time (ZT) point. 

### 2.5. Measurement of Blood Lead Level, Serum Creatinine Level and Daily Urine Amount

For confirmation of chronic lead intoxication in rats, blood lead levels were measured after the recording of the sleep–wake cycle. The rats were sacrificed at week six, and the blood sample was collected from the inferior vena cava to determine the concentration of blood lead and serum creatinine [28]. The 24 h urine sample was collected from metabolic cages for the measurement of urine amount [28]. Blood lead levels were measured by a Zeeman-effect graphite furnace atomic absorption spectrometry (Perkin–Elmer 5100 PC with AS 60 autosampler, EquipX, San Jose, CA, USA) in the same laboratory [8,9]. For intralaboratory quality controls, with the use of commercial standard materials (Betherning Institute, Bio-Rad, Hercules, CA, USA), all coefficients of variation were less than 3% for measurements at high (70.5 to 82.7 μg/dL) and medium levels (37.1 to 45.3 μg/dL), and were less than 5% for those at low levels (5.6 to 8.9 μg/dL). The detection limit of this instrument was 0.1 μg/dL. In addition, our laboratory has been participating in the interlaboratory blood-lead testing program in Taiwan [8,9]; the results of our measurements are all within the reference ranges, indicating that our blood lead measurements are accurate.

### 2.6. Analysis of Clock Gene Expression

The rats were anesthetized intraperitoneally with an injection of Zoletil^®^ 50, the brain was then rapidly removed under visual inspection and placed on a piece of gauze moistened with ice-cold 0.9% saline. The hypothalamus was routinely dissected and stored at −80 °C for further investigating the clock gene expression.

Eight rats (four in the lead-exposure group and four in the control group) at each pre-determined ZT point (ZT2, ZT6, ZT10, ZT14, ZT18, and ZT22) were scarified for analyzing whether expressional changes of clock genes took place during chronic lead exposure. Three clock genes, including the rat period 1 gene (rPer1), rat period 1 gene (rPer2) and rat brain and muscle ARNT-like 1b gene (rBmal1b) were selected for analysis of mRNA expression. In brief, the tissue of hypothalamus was immediately transferred to RNA stabilization reagent kits, RNAlater^®^ (Ambion, Valencia, CA, USA). Total RNA was extracted and purified by the spin column-based method (RNeasy Mini Kit^®^, 74104, QIAGEN, Hilden, Germany) following retrotranscription to cDNA with RevertAid First Strand cDNA Synthesis Kit^®^ (K1622, Waltham, MA, USA). All procedures were according to the manufacturer’s protocol as per our previous reports [28,30]. Subsequently, the cDNA was employed for TaqMan real-time quantitative PCR analysis by StepOnePlus™ Real-Time PCR System (Applied Biosystems, Foster City, CA, USA). The sequence of probe and primers used in this study were as follows: rPer1 (probe: 5′-CTTCAGCC-3′, forward: 5′-TGCTTTAGATCGGCAGTGGT-3′, reverse: 5′-GTGGGCTTGACACCTCTTCT-3′); rPer2 (probe: 5′-CTTCAGCC-3′, forward: 5′-GCTGGAGGACTACTTTGCATTT-3′, reverse: 5′-CCAGTGGCAAGAGTCAAAGC-3′) and *rBmal1b* (probe: 5′-TCCTCTCC-3′, forward: 5′-TGTCTGGAGTCCCTCCATTT-3′, reverse: 5′-AGTACGCCTCCCCCTGAT-3′).

Real-time quantitative PCR was performed in the TaqMan^®^ Universal PCR Master Mix (2X) (Applied Biosystems). All reactions were carried out in a 20 μL final volume containing 300 nM of each primer, 250 nM of the probe, 1 μL of TaqMan^®^ 20× probe/primer assay ix, and 1 μL of cDNA for the optimal performance. The “comparative Ct method” was applied to relatively quantify target genes expressed. The relative changes were determined from the Ct values of samples in chronic lead-exposure and control groups, and normalized to an endogenous housekeeping gene, β-actin.

### 2.7. Statistical Analysis

The concentration of blood lead and serum creatinine, urine output and the architecture of the sleep–wake cycle were presented as mean ± standard deviation. We assumed the difference of blood lead level between exposure and control groups was 10 μg/dL, and difference of EEG between the sleep–wake cycle was 15%, then the power calculated would be more than 0.6 when *n* = 6 for each group of rats. The quantitative gene expressions of *rPer1*, *rPer2*, and *rBmal1b* between the lead-exposure and control groups were presented as the ratio of mRNA levels of clock genes to the housekeeping gene, β-actin. Three consecutive ZT points and twelve ZT points for light and dark periods were grouped for statistical analysis. Differences in the variables between lead-exposure and control groups were conducted using a Mann–Whitney U test. The statistical analysis was performed using the IBM SPSS statistics version 22.0 (IBM, Armonk, NY, USA). A *p* < 0.05 was considered statistically significant.

## 3. Results

### 3.1. The Blood Lead Level and Biochemical Assay

The body weight, blood level of lead, serum level of creatinine, and daily urine amount in lead-exposure and control animals are presented in Table 1. There was no difference in body weight between two groups. Compared to the normal control rats, increased urine output was noted in lead-treated animals. Additionally, significant increases in blood level of lead (15.30 ± 3.22 μg/dL; ranged from 12.5 to 21.3 μg/dL) and serum level of creatinine were found in the lead-exposure group.

### 3.2. Alterations of Sleep–Wake Activities in Rats with Chronic Lead Poisoning

**Figure 1:**
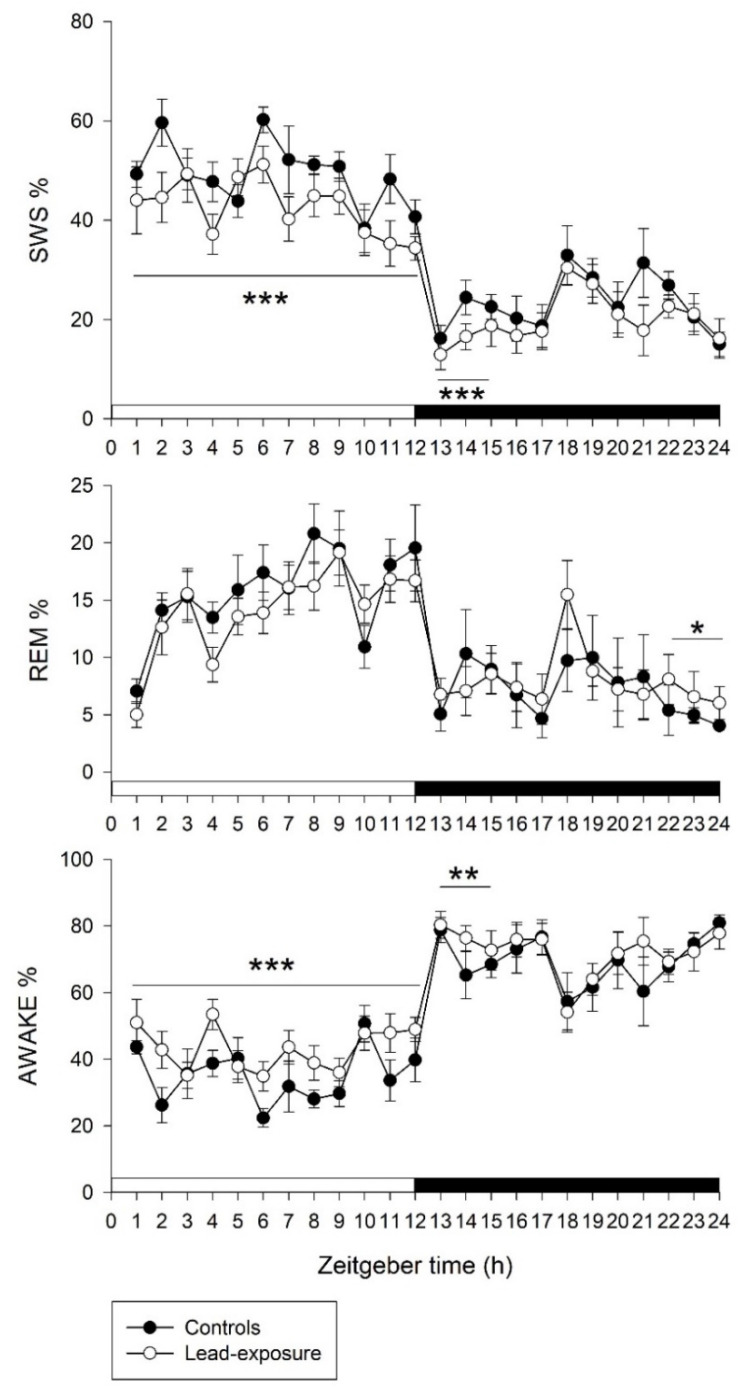
A 24 h panel of sleep–wake cycle between lead-exposure and control rats. Values on y-axis represent percentage of each sleep stage. Values on x-axis represent circadian Zeitgeber time points. SWS, slow wave sleep; REM, REM sleep; awake, wakefulness period. Data are represented as mean ± standard deviation. The two groups within 12h-interval or 3h-interval were compared by using a Mann–Whitney U test. * *p* < 0.05, ** *p* < 0.01 and *** *p* < 0.001 versus the control group.

According to EEG and EMG recordings, the sleep–wake cycle in animals with lead exposure and controls is shown in Figure 1. Comparison between lead-exposure and control groups in a 12 h interval of light and dark periods revealed that rats with chronic lead exposure had a significant decrease in SWS (42.68 ± 2.29% versus 48.72 ± 1.87%, *p* < 0.001) and increase in wakefulness (43.17 ± 2.80% versus 35.82 ± 2.53%, *p* < 0.001) compared with controls in the light period. Otherwise, the REM sleep in the light period did not show statistically significant between the lead-exposure and control groups (14.14 ± 1.61% versus 15.46 ± 1.77%, *p* = 0.141). However, there was no significant difference between the lead-exposure and control groups in the SWS, REM sleep and wakefulness in the dark period.

We further evaluated the sleep–wake architecture during the dark period in a 3 h interval. Based on statistical analysis, a significant decrease in SWS (16.10 ± 2.2 versus 21.10 ± 1.3, *p* < 0.001) and an increase in wakefulness (76.50 ± 2.6% versus 70.80 ± 2.0%, *p* = 0.005) were found at the ZT13-15 point in lead-exposure animals compared with controls. Moreover, a significant increase in REM sleep (6.80 ± 1.60% versus 4.79 ± 1.28%, *p* = 0.015) was noted at the ZT22-24 point in the lead-exposure group compared with the control group.

### 3.3. Alterations of the Clock Gene Expression in Rats with Chronic Lead Poisoning

**Figure 2:**
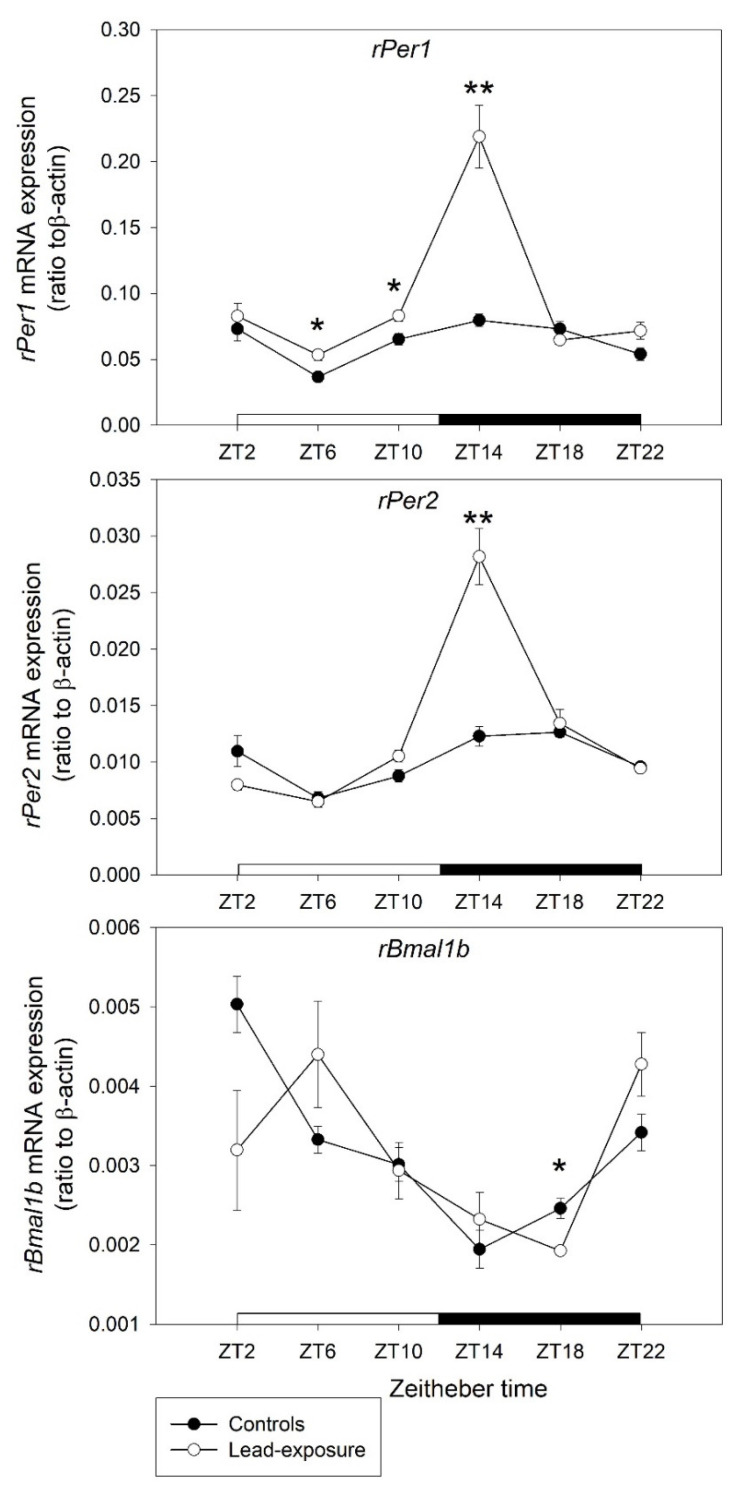
Clock gene mRNA expression in the hypothalamus between lead-exposure rat and control rats. Values on y-axis represent normalized as ratio of mRNA levels of clock genes to the housekeeping gene, β-actin. Values on x-axis represent circadian Zeitgeber time (ZT) points. Data are represented as mean ± standard deviation. * *p* < 0.05 and ** *p* < 0.01 versus the control group in Mann–Whitney U test.

The expressional changes of three clock genes (*rPer1*, *rPer2*, and *rBmal1b*) at each ZT points in the tissue from hypothalamus are shown in Figure 2. In the rats with chronic lead exposure, there was a significant upregulation of *rPer1* expression compared with control animals at ZT6 (0.0533 ± 0.0041 versus 0.0366 ± 0.0035, *p* = 0.022), ZT10 (0.0830 ± 0.0041 versus 0.0652 ± 0.0042, *p* = 0.030) and ZT14 (0.2190 ± 0.0239 versus 0.0796 ± 0.0047, *p* = 0.008). The expression of *rPer2* in lead-exposure animals had significant upregulation compared with controls (0.0282 ± 0.0025 versus 0.0123 ± 0.0009, *p* = 0.0046) at the ZT14 point. Otherwise, there was a significant downregulation of *rBmal1b* expression in lead-exposed rats at the ZT18 point (0.0019 ± 0.0000 versus 0.0025 ± 0.0001, *p* = 0.0195).

## 4. Discussion

Based on a clinically relevant experimental model, the present study demonstrated that rats with lead exposure for five weeks decreased SWS and increased wakefulness in the whole light period (ZT1 to ZT12) and the early dark period (ZT13 to ZT15) that was followed by a rebound of REM sleep at the end of the dark period (ZT22 to ZT24). The disturbance of sleep–wake cycle was associated with changes in clock gene expression that were characterized by upregulation of *rPer1* and *rPer2* and feedback repression of *rBmal1b*. The average blood level of lead-exposure animals was 15.30 ± 3.22 μg/dL, which ranged from 12.5 to 21.3 μg/dL. The exposure blood lead level in relation to human is considered as the low-to-medium level, which is thought to resemble the blood lead levels in chronic lead-exposed workers in Taiwan (the mean blood lead level was around 25 μg/dL) according to our previous human studies [8,9,37]. Our major findings indicate that chronic lead exposure predominantly disrupts homeostasis of the sleep–wake cycle in rats with a subsequent compensation by circadian process. In addition, increases in serum creatinine level and daily urine output may suggest chronic lead exposure may damage the renal interstitium that cause interstitial nephritis in rats, leading to impaired ability to concentrate urine and nocturnal polyuria [38,39,40].

The CNS is particularly susceptible to lead neurotoxicity that can result in a variety of neurological disorders [7,14,19]. It has been reported that lead exposure disrupts the temporal organization of behavioral patterns, circadian rhythm of locomotor activity and the sleep–wake cycle [23,25]. Shorter sleep duration and excessive daytime sleepiness may represent an unrecognized adverse consequence of chronic lead exposure in youth [21,22]. In the present study, we found that animals with chronic lead poisoning decreased SWS and increased wakefulness in the light period and the early part of the dark period. The homeostatic pressure built up in response to decreased SWS at the same periods. Evidently, the duration of deep sleep in the whole light/dark period was reduced under chronic lead neurotoxicity. The SWS, which is the major component of non-rapid eye movement (NREM) sleep, has a strong link to homeostatic process [41]. Decreased SWS and hyperarousal may lead to irritability, fatigue, sleep deprivation, as well as increased daytime sleepiness [42]. We noted especially that rebound promotion of REM sleep occurred at the end of the dark period in the chronic lead-exposure rats. The phenomenon of daytime sleepiness with REM sleep rebound decreased after SWS and sleep deprivation, which may be related to the activated compensatory mechanism of circadian and homeostatic control process in the SCN of hypothalamus [43,44]. 

The dysregulation of sleep homeostasis can be compensated by the circadian process, which is a well-documented phenomenon in humans with chronic primary insomnia [45]. This circadian modulation is weaker and less clear in rodents than in humans [46]. However, under constant homeostatic sleep pressure, an endogenous circadian modulation of the duration of waking and NREM sleep has been observed in rats [46,47]. Whereas the active promotion of REM sleep was observed in response to a decrease in SWS and sleep deprivation at the end of the dark period, there was no compensatory increase in SWS in the subsequent dark period in the chronic lead-exposure rats. SWS deficits might mean that the integrity of homeostatic regulatory mechanism and its related restorative functions are impaired [48]. The abnormal molecular circuitry of circadian clocks through the expressional changes of clock genes might play an important role in the chronic lead-exposure rats [49]. 

*rPer1* and *rPer2* are members of the period family of genes [50,51,52]. The genes in this family encode components of the circadian rhythms of locomotor activity, metabolism, sleep–wake cycle, and behavior [49,51,53]. The SCN, the primary circadian pacemaker in the mammalian brain, is composed of subgroups of cells that are different in circadian phase and oscillation magnitude of the *rPer1* and *rPer2* genes [50,51]. Quantitative in situ hybridization of *rPer1* and *rPer2* mRNAs showed a robust circadian rhythm in the SCN with a characteristic peak/trough profile in each gene [54,55]. The peak level of *rPer1* mRNA was presented in the daytime and that of *rPer2* mRNA was at the transition time of day to night in both light–dark and constant dark conditions [54,55]. Prolonged light exposure during daytime positively modulates daily levels of *Per1* and *Per2* mRNA in the SCN [56]. BMAL1 was suggested to be a partner of circadian locomotor output cycles kaput (CLOCK) and a component of a molecular feedback loop of circadian oscillation [49,52]. In the classic view, CLOCK and BMAL1 increase the transcription of Period (Per), Cryptochrome (Cry), and other clock-controlled genes during the daytime [49,52,53]. The levels of Per and Cry proteins increase during the night, after which they dimerize and translocate to the nucleus to repress CLOCK–BMAL1-mediated transcription [49,52,53].

In the present study, we found that a significant upregulation of *rPer1* expression started at ZT6 to ZT14 with a heightened expressional level at Z14 point in the chronic lead-exposure rats. The expression of *rPer2* also was highly upregulated at the ZT14 point. On the other hand, there was a significant downregulation of *rBmal1b* expression at the ZT18 point in chronic lead-exposure animals. The expression of *rPer1* and *rPer2* was heightened during the early dark period. This phenomenon was contrary to the normal circadian pattern that would be found in the daytime with the peak level of *rPer1* and *rPer2* mRNAs [54,55,56]. Then, the abnormally high expression of *rPer1* and *rPer2* during the early dark period inhibited *rBmal1b* expression at the late stage of the dark period from the negative feedback loop. The abnormal expression of *rPer1*, *rPer2*, and *rBmal1b* may result in the disturbance of the circadian sleep–wake cycle and disruption of sleep homeostasis in chronic lead-exposure rats. Thus, we suggested that chronic lead exposure may exert toxic effects on the cells of SCN in the hypothalamus that result in abnormal changes of clock gene expression in rats. However, further studies are warranted to address these issues.

The molecular and cellular mechanism of lead neurotoxicity to the cells of SCN is still unclear. Several mechanisms have been proposed, such as ion mimicry, mitochondrial dysfunction, redox imbalance, and neuroinflammation [14]. The homeostatic dysregulation following lead neurotoxicity may be based on the similarity of ionized lead to calcium [57]. Lead exposure can disrupt calcium homeostasis caused by the abnormal calcium transportation [58]. Recent evidence has suggested that the toxic effects of lead might be brought on by occupying calcium-binding sites on calmodulin [14,17]. These effects might influence circadian regulation of voltage-dependent calcium channel activity that plays an important role in maintaining rhythmic clock gene expression associated with SCN oscillators [59]. Moreover, both adenosine and nitric oxide (NO) are known for their role in sleep homeostasis [60]. Adenosine causes a hyperpolarization of membrane potential and a reduction in postsynaptic potentials in the hypothalamus, which are calcium dependent through glutamate receptors [61]. The abnormal expression of glutamate receptor in the hippocampus and cerebral cortex might be involved in the sleep–wake homeostasis through wake-promoting glutaminergic activators [62] and sleep-promoting glutaminergic inhibitors [63]. In addition, the homeostatic dysregulation following lead neurotoxicity may be involved in NO synthesis, which has a role in homeostasis in the basal forebrain of rats [64]. Lead exposure has been shown to inhibit NO synthase (NOS) activity in capillary and synaptosomal fractions of mouse brains [65]. This evidence showed that the adenosine, inducible NOS, and NO production may play a crucial role in the temporal and spatial sequence of sleep homeostatic cascade [60].

The homeostatic dysregulation following lead neurotoxicity could be caused by an imbalance between acetylcholine and dopamine. It has been reported that acetylcholine in the preoptic area and anterior hypothalamus is important in regulation of homeostasis of core body temperature during sleep [66]. Lead exposure might induce the inhibition of acetylcholine receptor through enhancement of extracellular calcium concentrations [67]. Lead poisoning and some psychostimulants, such as amphetamine and modafinil, can act on dopamine-rich brain areas, including the striatum and the nucleus accumbens, that have a strong influence on homeostasis through a wake-promoting effect [68,69].

Furthermore, lead poisoning may cause a neuroinflammatory response that involves the production and release of inflammatory cytokines, augmented oxidative stress, and diminished antioxidant activity, resulting in neuronal injury or neuronal loss in the CNS [14]. The neural cell adhesion molecules (NCAM) and cytokines might also play a role in the pathogenesis of dysregulation of homeostatic process following lead neurotoxicity. The NCAM is a potential early target in the neurotoxicity of lead [70]. NCAM is involved in activity-dependent development and synaptic plasticity, which occurs during homeostatic processes [71], not only in the regulation of wakefulness and NREM sleep but also in the homeostasis of REM sleep [72]. The T cells are critical functional targets of lead immunotoxicity. A single exposure to lead might activate CD4(+) T cells [73] and lead to the production of cytokines through CD40 and disrupt the sleep–wake regulatory circuit [74].

Disturbed sleep homeostasis is a common problem in occupational health. Most issues have focused on chronic sleep deprivation due to rotating shift work. In the present study, we demonstrated that chronic lead exposure predominantly disrupts homeostasis of the sleep–wake cycle, resulting in sleep deprivation. We suggest that sleep disturbance can be an unrecognized consequence in individuals with chronic lead exposure. Exposure to even low levels of lead can cause damage over time, especially in children [75,76]. The greatest risk is to brain development, where irreversible neuropsychological and functional decline can occur in later life [7,75,76]. Moreover, chronic low levels of lead exposure can damage the renal, hematological, cardiovascular, and nervous systems in both children and adults [4,75,76,77,78]. We thus emphasize that the focus of environmental and occupational medicine on lead neurotoxicity should be extended from acute lead poisoning to unrecognized chronic lead exposure.

There are some limitations to the present study. First, we selected the common circadian genes, including *rPer1*, *rPer2,* and *rBmal1b*, to reveal the role of altered expressions of clock genes under chronic lead exposure in rats. Nevertheless, the expressional changes of other circadian rhythm genes, such as Clock, REV-ERBα, and CRY, are advantageous to understand the crucial relationship between the chronic lead intoxication and circadian rhythm genes. Second, the mechanism of lead toxicity in changes of clock gene expression is unclear. Understanding the molecular and cellular mechanisms of lead neurotoxicity is required to advance studies. Third, the SCN cell is relatively small and the investigation of changes in clock genes in the SCN is not easy. Quantitative in situ hybridization of mRNAs of clock genes may be considered. Fourth, the exposure level of lead in the animal studies is a limitation because rats have a short lifespan compared to humans. In the present study, the chronic lead-exposure level of rats achieved to 15 μg/dL that needed to use high concentration of lead water, 250 ppm, which was much higher than human drinking water. Overall, further studies are warranted to address these issues.

## 5. Conclusions

The present study indicates that a chronic low-to-medium level of lead exposure has a potential and negative impact on the sleep–wake cycle in rats that predominantly disrupts sleep homeostasis. The disruption of sleep homeostasis was associated with a toxic effect of lead on the clock gene expression in the hypothalamus. Since environmental and occupational exposure is a commonly known cause of lead poisoning, careful monitoring of the toxicity and blood level of lead in the population with a high risk of chronic lead exposure is particularly important.

## Figures and Tables

**Table 1:** Body weight, urine amount, and biochemical data in lead-exposure and control animals.

Term	Lead-Exposure Group (*n* = 6)	Control Group (*n* = 6)	*p*
Body weight (g)	406.6 ± 25.8	412.1 ± 20.7	0.925
Blood lead (μg/dL)	15.30 ± 3.22	0.90 ± 0.10	<0.001
Serum creatinine (mg/dL) Daily urine amount (mL)	0.39 ± 0.18 22.3 ± 5.1	0.23 ± 0.06 15.8 ± 4.9	0.039 0.017

Values are expressed as mean ± standard deviation.

## Data Availability

The data used to support the findings of this study are included within the article.
